# Retraction Note: Assessment of the influence of whole body vibration on Cochlear function

**DOI:** 10.1186/s12995-017-0162-9

**Published:** 2017-06-29

**Authors:** Seyyed-Ali Moussavi-Najarkola, Ali Khavanin, Ramazan Mirzaei, Mojdeh Salehnia, Mehdi Akbari

**Affiliations:** 1 Department of Occupational Health, School of Medical Sciences, Tarbiat Modares University (TMU), Tehran, Iran; 2 Department of Occupational Health, Health promotion research center, Zahedan University of Medical Sciences (ZUMS), Zahedan, Iran; 3 Department of Anatomical Sciences, School of Medical Sciences, Tarbiat Modares University (TMU), Tehran, Iran; 4 Department of Audiology, School of Rehabilitation, Iran University of Medical Sciences (IUMS), Tehran, Iran

## Retraction Note

The Editors-in-Chief are retracting this article [1] as it has already been published in *In Vitro* *Cellular & Developmental Biology – Animal* [2]. The authors do not agree with this retraction.

## References

[1] Moussavi-Najarkola S, Khavanin A, Mirzaei R, Salehnia M, Akbari M (2012). Assessment of the influence of whole body vibration on Cochlear function. Journal of Occupational Medicine and Toxicology.

[2] Moussavi-Najarkola S, Khavanin A, Mirzaei R, Salehnia M, Akbari M (2012). Effects of whole body vibration on outer hair cells’ hearing response to distortion product otoacoustic emissions. In Vitro Cell Dev Biol Anim.

